# Supplementary material for: Sensory ASIC3 channel exacerbates psoriatic inflammation via a neurogenic pathway in female mice
Source: Nat Commun. 2024 Jun 20;15:5288. doi: 10.1038/s41467-024-49577-3 (PMC11190258; doi:10.1038/s41467-024-49577-3)
Supplement: Supplementary file 1 — Supplementary Information [file 41467_2024_49577_MOESM1_ESM.pdf]

Supplementary Information for

Sensory ASIC3 channel exacerbates psoriatic inflammation via a neurogenic pathway in female mice

Chen Huang[1–3,11], Pei-Yi Sun[4,11], Yiming Jiang[2,5,11], Yuandong Liu[6], Zhichao Liu[6], Shao-Ling Han[2], Bao-Shan Wang[2], Yong-Xin Huang[2], An-Ran Ren[2], Jian-Fei Lu[1,2], Qin Jiang[1,2], Ying Li[3], Michael X. Zhu[7], Zhirong Yao[4], Yang Tian[6], Xin Qi[1,2,*], Wei-Guang Li[2,8–10,*], Tian-Le Xu[1,2,10*]

[1]Department of Anesthesiology, Songjiang Hospital and Songjiang Research Institute, Shanghai Key Laboratory of Emotions and Affective Disorders, Shanghai Jiao Tong University School of Medicine, Shanghai 201600, China
[2]Department of Anatomy and Physiology, Shanghai Jiao Tong University School of Medicine, Shanghai 200025, China
[3]Basic Medicine Experimental Teaching Center, Shanghai Jiao Tong University School of Medicine, Shanghai 200025, China
[4]Department of Dermatology, Xinhua Hospital, Institute of Dermatology, Shanghai Jiao Tong University School of Medicine, Shanghai 200092, China
[5]Department of Otorhinolaryngology, Renji Hospital, Shanghai Jiao Tong University School of Medicine, Shanghai 200127, China
[6]Shanghai Key Laboratory of Green Chemistry and Chemical Processes, Department of Chemistry, School of Chemistry and Molecular Engineering, East China Normal University, Shanghai 200241, China
[7]Department of Integrative Biology and Pharmacology, McGovern Medical School, The University of Texas Health Science Center at Houston, Houston, TX 77030, USA
[8]Department of Rehabilitation Medicine, Huashan Hospital, Institute for Translational Brain Research, State Key Laboratory of Medical Neurobiology and Ministry of Education Frontiers Center for Brain Science, Fudan University, Shanghai 200032, China
[9]Ministry of Education-Shanghai Key Laboratory for Children's Environmental Health, Xinhua Hospital Affiliated to Shanghai Jiao Tong University School of Medicine, Shanghai 200092, China
[10]Shanghai Research Center for Brain Science and Brain-Inspired Intelligence, Shanghai 201210, China
[11]These authors contributed equally: Chen Huang, Pei-Yi Sun, Yiming Jiang.
*Correspondance: xin597454490@163.com (X.Q.), liwg@fudan.edu.cn (W.-G.L.); xu-happiness@shsmu.edu.cn (T.-L.X.).

Supplementary Information provided in this file:

Supplementary Fig. 1 Characterization of abnormal increase of keratinocytes upper basal layer in psoriatic model.
Supplementary Fig. 2 Efficacy of selective deletion of ASIC3 in nociceptors in *Nav1.8*^{Cre}*::Asic3*$^{flox/flox}$ mice.
Supplementary Fig. 3 Genetic knockdown of ASIC3 in peripheral nervous system attenuates immune responses of psoriasis.
Supplementary Fig. 4 Conditional rescue of ASIC3 expression in nociceptors restores immune responses of psoriasis.
Supplementary Fig. 5 Co-expression of ASIC3 and TRPV1 in nociceptors and skin afferents in *Asic3-myc* mice.
Supplementary Fig. 6 *Asic3* KO mice exhibit a similar improvement in psoriatic inflammation as nociceptor ablation.
Supplementary Fig. 7 Effects of BoNT/A or bicuculline on CGRP release in DRG neurons.
Supplementary Fig. 8 CGRP mediates DC-derived IL-23 production in DRG-BMDC cocultures.
Supplementary Fig. 9 CGRP increases proliferation of HaCaT keratinocytes.
Supplementary Fig. 10 Psoriatic skin exhibits acidosis.
Supplementary Fig. 11 Psoriatic skin exhibits altered lipid profiles in lesional skin.
Supplementary Fig. 12 Variable involvement of different LPC molecular species in psoriatic inflammation.
Supplementary Fig. 13 Uncropped images of Western blots shown in Supplementary Fig. 3c.

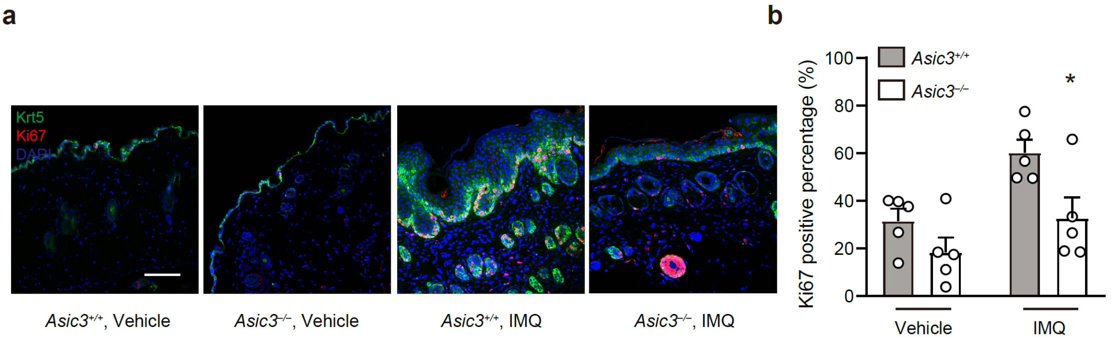

Supplementary Fig. 1 Characterization of abnormal increase of keratinocytes upper basal layer in psoriatic model. a Ki67 exhibited sparce expression in epidermal basal layer of both *Asic3*$^{+/+}$ and *Asic3*$^{-/-}$ mice treated by vehicle. IMQ induced widespread expression of Ki67 in epidermis of lesioned skin in *Asic3*$^{+/+}$, but not *Asic3*$^{-/-}$, mice. Scale bar, 100 µm. **b** Quantification of percentage of Ki67 positive cells in Krt5 positive epidermal cells. IMQ: $F_{(1,16)}$ = 9.778, $^{*}p$ = 0.0173 < 0.05, *Asic3*$^{-/-}$ vs. *Asic3*$^{+/+}$, two-way ANOVA. n = 5 mice per group. Summary data are mean ± SEM.

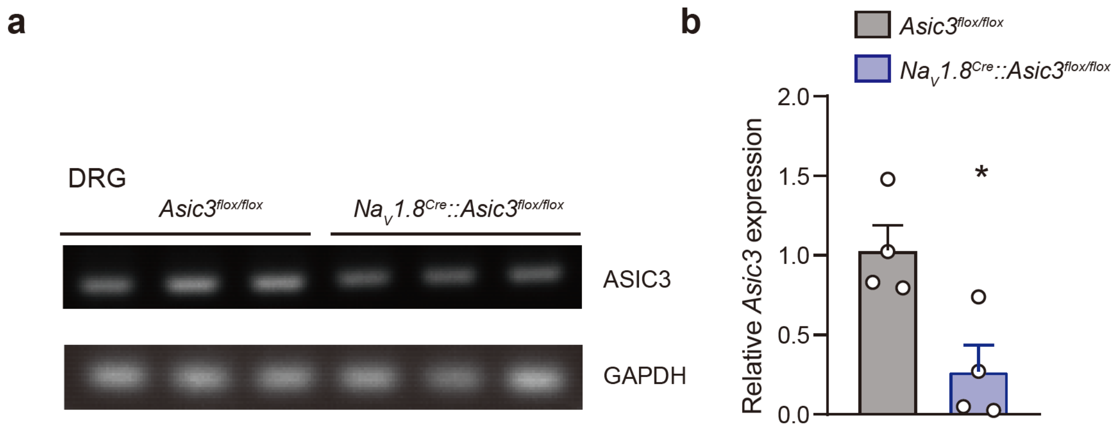

Supplementary Fig. 2 Efficacy of selective deletion of ASIC3 in nociceptors in *Na$_V$1.8^{Cre}::Asic3$^{flox/flox}$* mice. a Representative agarose gels showing ASIC3 mRNA expression in DRGs of *Asic3$^{flox/flox}$* mice and *Na$_V$1.8^{Cre}::Asic3$^{flox/flox}$* mice, determined by RT-PCR. **b** Relative Asic3 expression by RT-qPCR in DRGs of *Asic3$^{flox/flox}$* mice and *Na$_V$1.8^{Cre}::Asic3$^{flox/flox}$* mice. $^{*}p = 0.0157 < 0.05$, *Asic3$^{flox/flox}$* vs. *Na$_V$1.8^{Cre}::Asic3$^{flox/flox}$*, two-tailed unpaired Student's t test. n = 4 mice per group. Summary data are mean ± SEM.

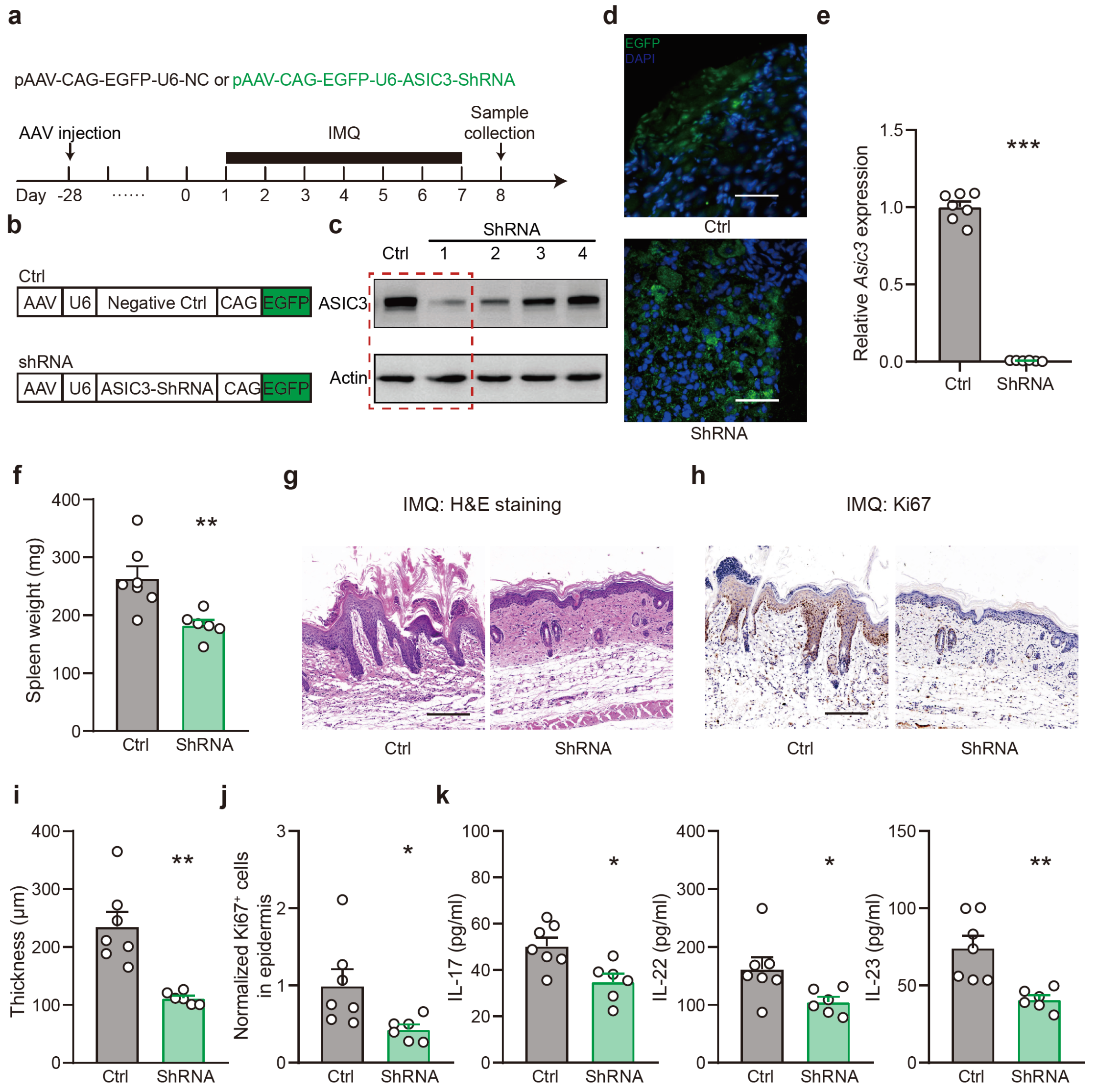

Supplementary Fig. 3 Genetic knockdown of ASIC3 in peripheral nervous system attenuates immune responses of psoriasis. **a** Schematic protocol of ASIC3 knockdown in peripheral neurons and induction of psoriatic skin inflammation. **b** Schematics of control and ASIC3-ShRNA constructs. **c** Efficiency of knockdown by different ASIC3-ShRNA constructs evaluated by western blotting using HEK-293 cells transfected with *mAsic3*. **d** Representative images of GFP (green) expression in DRG slices that received either AAV-CAG-eGFP-U6-ASIC3-ShRNA (ShRNA) or AAV-CAG-EGFP-U6-NC (Ctrl). DAPI (blue) was used to label nuclei. Scale bar, 100 μm. **e** Efficacy of ASIC3 knockdown in peripheral neurons, determined by RT-qPCR. Data were normalized to that of the control DRG tissues. $^{***}p < 0.001$, Ctrl vs. ShRNA, two-tailed unpaired Student's *t* test. **f** Spleen weight after psoriasis modeling in Ctrl mice and mice with ASIC3 knockdown in peripheral nerves. $^{**}p = 0.0067 < 0.01$, Ctrl vs. ShRNA, two-tailed unpaired Student's *t* test. **g** Representative H&E staining of lesional skin in Ctrl and mice with ASIC3 knockdown in peripheral nerves. Scale bar, 200 μm. **h** Representative Ki67 staining in lesional skin. Scale bar, 200 μm. **i** Quantification of

epidermal thickness in (**g**). $^{**}p = 0.0026 < 0.01$, Ctrl vs. ShRNA, two-tailed unpaired Student's *t* test. **j** Quantification of Ki67^{+} cells after psoriasis modeling. $^{*}p = 0.0373 < 0.05$, Ctrl vs. ShRNA, two-tailed unpaired Student's *t* test. **k** Psoriasis-related cytokine IL-17, IL-22 and IL-23 protein expression in lesional skin in Ctrl and mice with ASIC3 knockdown in peripheral nerves. IL-17: $^{*}p = 0.0103 < 0.05$; IL-22: $^{*}p = 0.0341 < 0.05$; IL-23: $^{**}p = 0.0047 < 0.01$, Ctrl vs. ShRNA, two-tailed unpaired Student's *t* test. n = 7 and 6 for Ctrl and shRNA groups, respectively. Summary data are mean ± SEM.

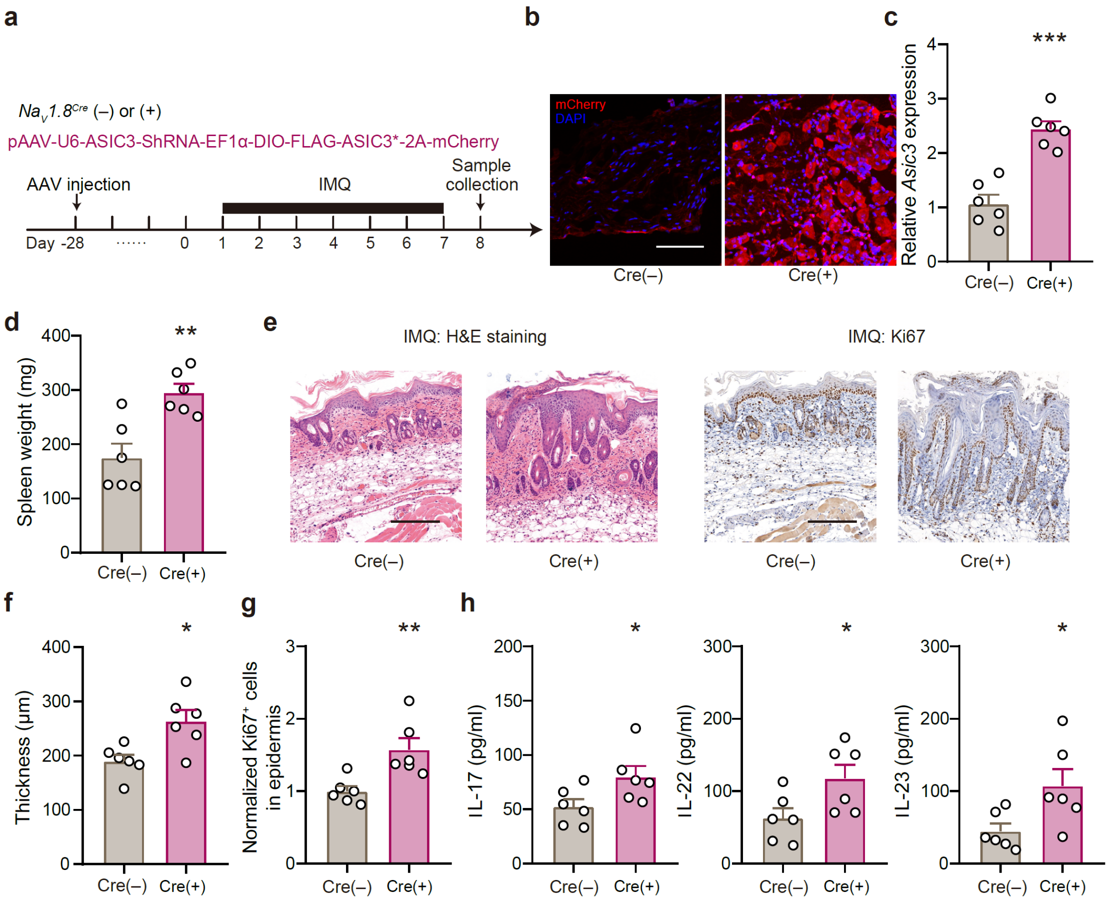

Supplementary Fig. 4 Conditional rescue of ASIC3 expression in nociceptors restores immune responses of psoriasis. a Schematic protocol of AAV injection to conditionally rescue ASIC3 expression in nociceptors. **b** Representative images of mCherry (red) expression in DRG slices from mice that received pAAV-U6-ASIC3-ShRNA-EF1α-DIO-FLAG-ASIC3*-2A-mCherry. DAPI (blue) was used to label nuclei. Scale bar, 100 μm. **c** Relative Asic3 expression determined by qRT-PCR in $Nav1.8^{Cre}$ (–) and (+) DRG tissues. $^{***}p < 0.001$, Cre(–) vs. Cre(+), two-tailed unpaired Student's *t* test. **d** Spleen weight of mice in (**c**). $^{**}p = 0.0029 < 0.01$, Cre(–) vs. Cre(+), two-tailed unpaired Student's *t* test. **e** Representative H&E staining and Ki67 staining of lesional skin from control, Cre(–), and conditional ASIC3 rescue, Cre(+), mice treated with IMQ. Scale bar, 200 μm. **f** Quantification of epidermal thickness in (**e**). $^{*}p = 0.0113 < 0.05$, Cre(–) vs. Cre(+), two-tailed unpaired Student's *t* test. **g** Quantification of $Ki67^{+}$ cells after psoriasis modeling. $^{**}p = 0.0073 < 0.01$, Cre(–) vs. Cre(+), two-tailed unpaired Student's *t* test. **h** Psoriasis-related cytokine IL-17, IL-22, and IL-23 protein expression in lesional skin of control and conditional ASIC3 rescue mice. IL-17: $^{*}p = 0.0468 < 0.05$; IL-22: $^{*}p = 0.0392 < 0.05$; IL-23: $p = 0.0348 < 0.05$, Cre(–) vs. Cre(+), two-tailed unpaired Student's *t* test. n = 6 mice per group. Summary data are mean ± SEM.

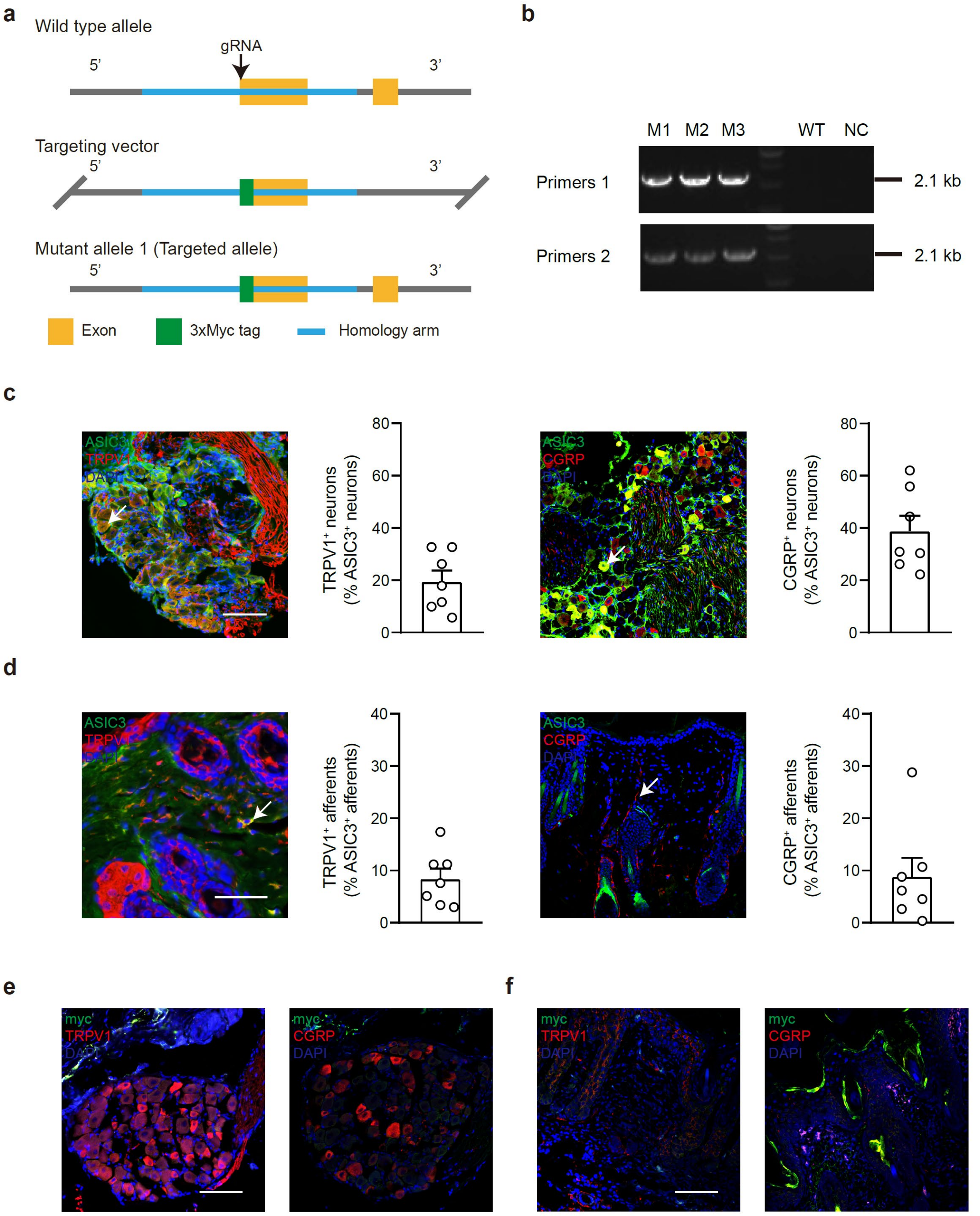

Supplementary Fig. 5 Co-expression of ASIC3 and TRPV1 in nociceptors and skin afferents in *Asic3-myc* mice. a Strategy of making *Asic3-myc* mice. *Asic3* gene is located on mouse chromosome 5, with ATG start codon in exon 1 and the TAG stop codon in exon 11. The 3×Myc tag was inserted downstream of the ATG start codon. **b** Myc expression in *Asic3-myc* mice detected by RT-PCR. Primers 1: 5' arm forward primer (F1): 5'-TTCCTGAAACTAAGGCCCGAGT-3'; 3' KI reverse primer (R1): 5'-CTCAGAGATGAGCTTCTGTTCCA-3'. Primers 2: 5' KI forward primer (F2): 5'-GAGCAGAAACTCATCTCTGAAGAAG-3'; 3' arm reverse primer (R2): 5'-

TTGGGACTTCTCCCTACACACCTA-3’. WT, wildtype mice; NC, no-template control. **c** Co-expression of ASIC3 and TRPV1 (*left*) or CGRP (*right*) in DRG. DRG slices from *Asic3*-myc mice were stained for myc (*green*), TRPV1 (*red*) or CGRP (*red*), and nuclei (by DAPI, *blue*). Shown are representative images (Scale bar, 100 μm) and quantification of percent TRPV1$^+$ or CGRP$^+$ cells that expressed ASIC3-myc (n = 7). **d** Co-expression of ASIC3 and TRPV1 (*left*) or CGRP (*right*) in skin afferents. Dorsal skin of *Asic3*-myc mice were stained for myc (*green*), TRPV1 (*red*) or CGRP (*red*), and nuclei (by DAPI, *blue*). Shown are representative images (Scale bar, 50 μm) and quantification of percent TRPV1$^+$ or CGRP$^+$ afferents that expressed ASIC3-myc (n = 7). For both c and d, white arrows indicate colocalized myc and TRPV1 or CGRP signals. **e** Representative images of DRG slices of WT mice stained for myc (*green*), TRPV1 (*red*) or CGRP (*red*), and nuclei (by DAPI, *blue*). Scale bar, 100 μm. **f** Representative images of dorsal skin slices of WT mice stained for myc (*green*), TRPV1 (*red*) or CGRP (*red*), and nuclei (by DAPI, *blue*). Scale bar, 100 μm.

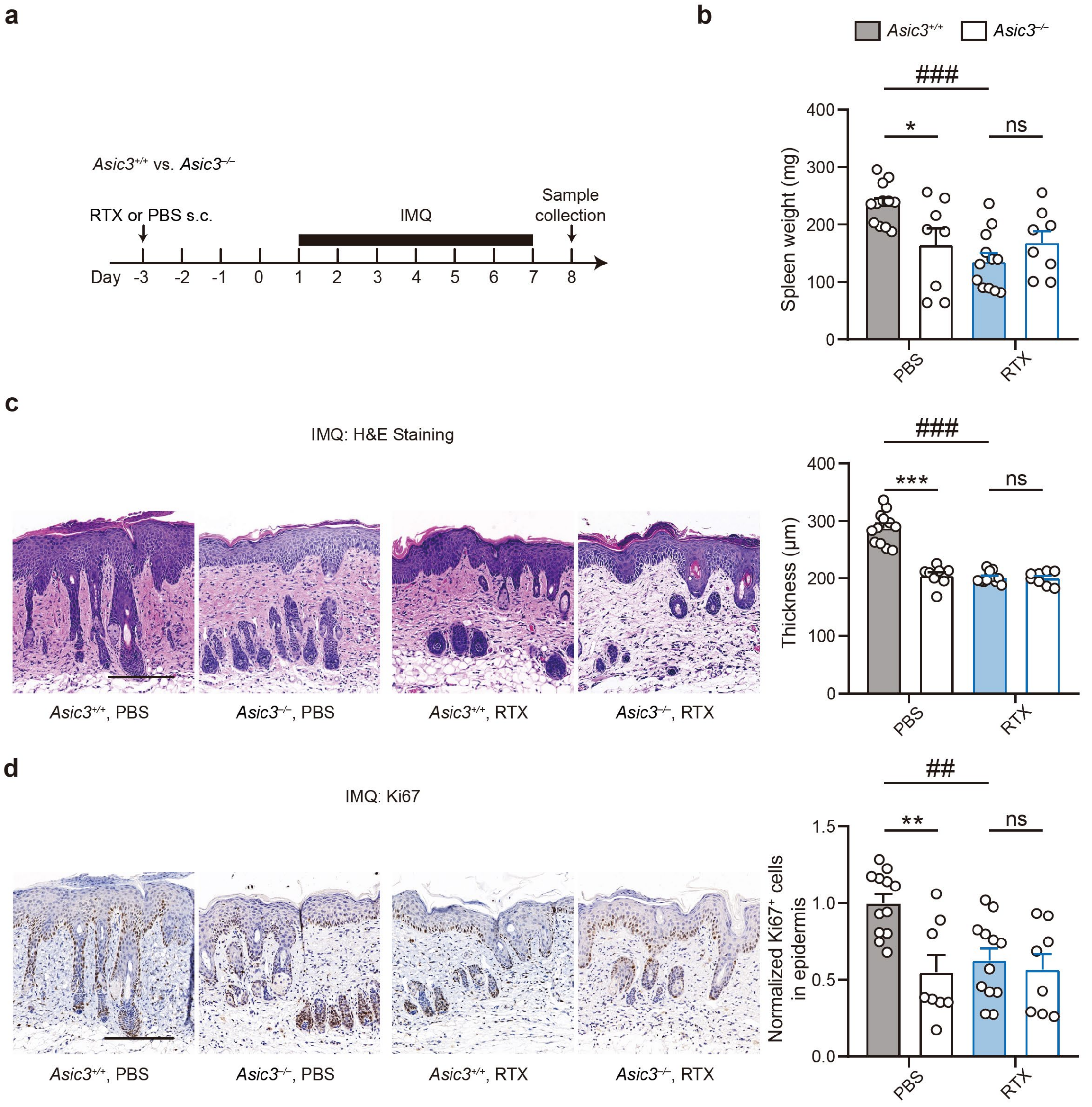

Supplementary Fig. 6 *Asic3* KO mice exhibit a similar improvement in psoriatic inflammation as nociceptor ablation. a Schematic diagram of timeline of silencing nociceptors and psoriasis modeling. **b** Spleen weight after psoriasis modeling of *Asic3*$^{+/+}$ and *Asic3*$^{-/-}$ mice without and with nociceptor ablation. PBS: $F_{(1,36)} = 1.167$, $^{*}p = 0.0153$; RTX: $p = 0.3641$, *Asic3*$^{-/-}$ vs. *Asic3*$^{+/+}$, two-way ANOVA. *Asic3*$^{+/+}$: $F_{(1,36)} = 7.331$, $^{\#\#\#}p = 0.0002 < 0.001$, RTX vs. PBS, two-way ANOVA. **c** Representative images of H&E staining (*left*) and quantification of epidermal thickness (*right*) in lesional skin. Scale bar, 200 μm. PBS: $F_{(1,36)} = 48.39$, $^{***}p < 0.001$; RTX: $p = 0.9828$, *Asic3*$^{-/-}$ vs. *Asic3*$^{+/+}$, two-way ANOVA. *Asic3*$^{+/+}$: $F_{(1,36)} = 54.74$, $^{\#\#\#}p < 0.001$, RTX vs. PBS, two-way ANOVA. **d** Representative images of Ki67 staining in lesional skin (*left*) and quantification of Ki67^{+} cells (*right*) for PBS and RTX-treated *Asic3*$^{+/+}$ and *Asic3*$^{-/-}$ mice after psoriasis modeling. Scale bar, 200 μm. PBS: $F_{(1,36)} = 9.065$, $^{**}p = 0.0012 < 0.01$; RTX: $p = 0.8529$, *Asic3*$^{-/-}$ vs. *Asic3*$^{+/+}$, two-way ANOVA. *Asic3*$^{+/+}$: $F_{(1,36)} = 4.324$, $^{\#\#}p = 0.0028 < 0.01$, RTX vs. PBS, two-way ANOVA. n = 12 and 8 for *Asic3*$^{+/+}$

and *Asic3*$^{-/-}$ groups, respectively. Summary data are mean ± SEM.

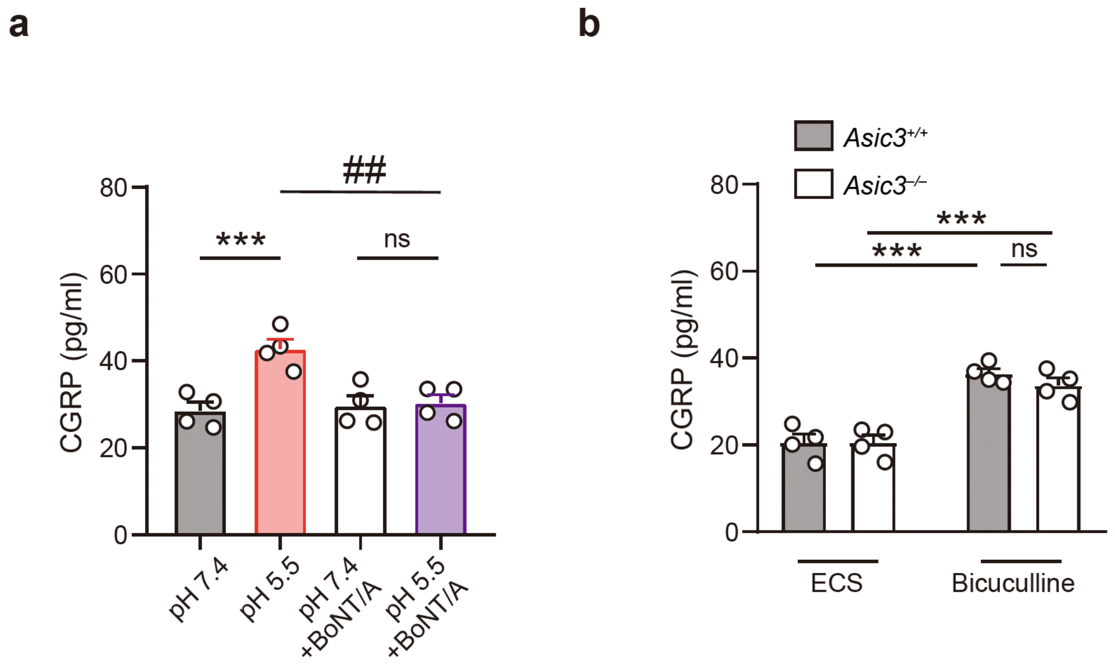

Supplementary Fig. 7 Effects of BoNT/A or bicuculline on CGRP release in DRG neurons. **a** BoNT/A blocked acid-induced CGRP release from DRG neurons. DRG neurons from *Asic3*$^{+/+}$ mice were incubated with BoNT/A, acid (pH 5.5), acid plus BoNT/A for 30 min and CGRP was measured in neuronal supernatant. $F_{(1,12)}$ = 12.40, $^{***}p$ = 0.0009 < 0.001, pH 7.4 vs. pH 5.5; p = 0.9724, pH 7.4+BoNT/A vs. pH 5.5+BoNT/A; $F_{(1,12)}$ = 7.216, $^{\#\#}p$ =0.0026 < 0.01, pH 5.5 vs. pH 5.5+BoNT/A, two-way ANOVA. n = 4 for each group. **b** GABA$_A$R antagonist bicuculline enhanced CGRP release in DRG neurons independently of ASIC3. DRG neurons from *Asic3*$^{+/+}$ and *Asic3*$^{-/-}$ mice were stimulated with bicuculline (10 μM) for 30 min and CGRP was measured in neuronal supernatant. $F_{(1,12)}$ = 0.6880, Bicuculline: p = 0.4705, *Asic3*$^{-/-}$ vs. *Asic3*$^{+/+}$; $F_{(1,12)}$ = 78.26, *Asic3*$^{+/+}$: $^{***}p$ < 0.001, ECS vs. Bicuculline, *Asic3*$^{-/-}$: $^{***}p$ = 0.0002 < 0.001, ECS vs. Bicuculline; two-way ANOVA.

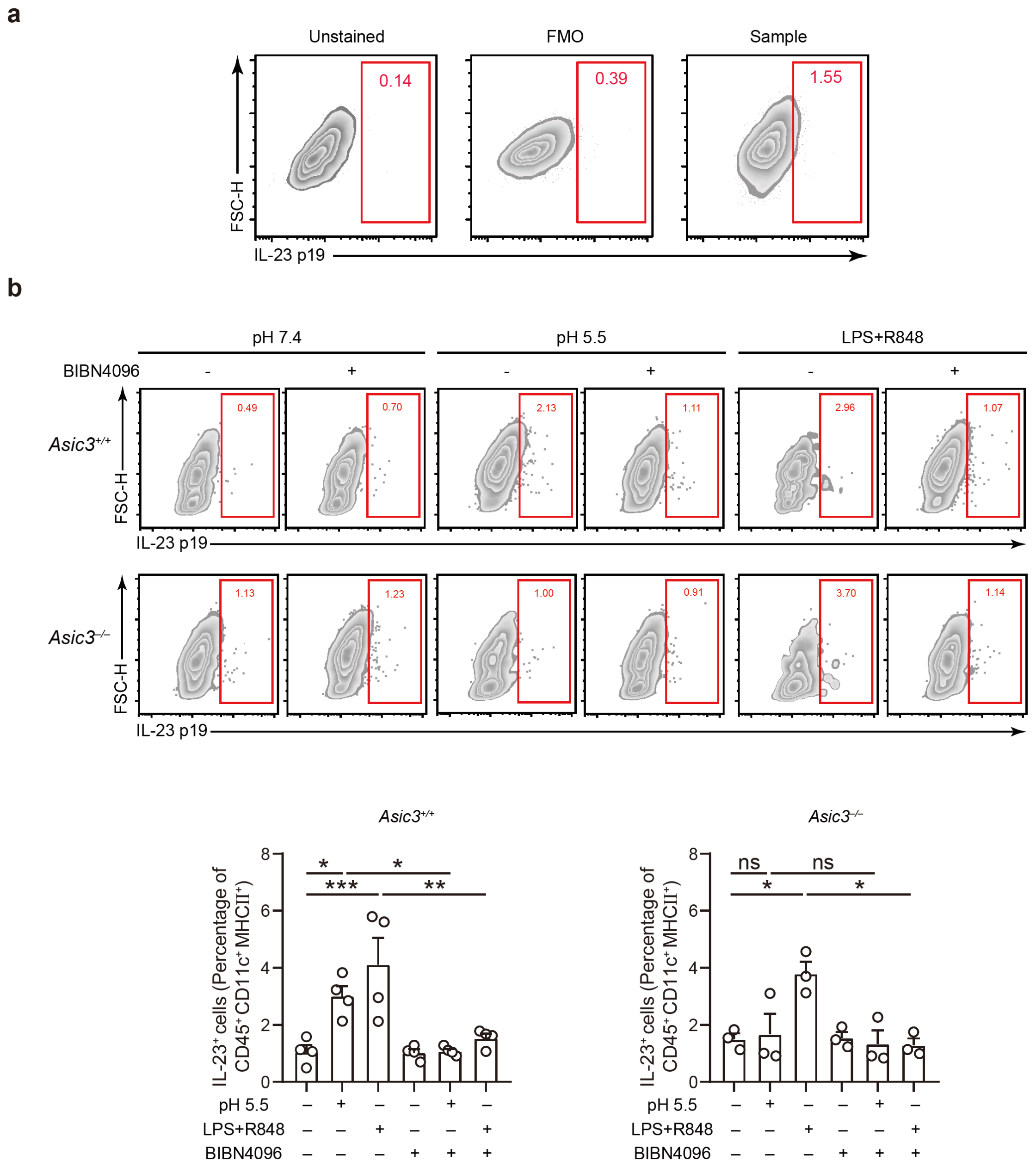

Supplementary Fig. 8 CGRP mediates DC-derived IL-23 production in DRG-BMDC cocultures. a Gating strategy for BMDCs cyto flowmetry**. b.** FMO control of IL-23 in cocultured BMDCs and DRG neurons. Dot plots of unstained, FMO control and a fully stained sample of cocultured IL-23^{+} BMDCs and DRG neurons. BMDC, bone marrow-derived dendritic cell; DRG, dorsal root ganglion; FMO, fluorescence minus one; FSC-H, forward scatter height. **c** Flow cytometric analysis of IL-23^{+} BMDCs in DRG-BMDC cocultures treated with ECS, acid (pH 5.5), or LPS plus R848 followed by BIBM4096 or PBS. *Asic3*$^{+/+}$: $^{*}p$ =0.0452 < 0.05, pH 7.4 vs. pH 5.5, $^{*}p$ =0.0482 < 0.05, pH 5.5 vs. pH 5.5+BIBN4096, $^{***}p$ =0.0009 < 0.001, pH 7.4 vs. LPS+R848, $^{**}p$ =0.0050 < 0.01, LPS+R848 vs. LPS+R848+BIBN4096; *Asic3*$^{-/-}$: p = 0.9997, pH 7.4 vs. pH 5.5, p = 0.9920, pH 5.5 vs. pH 5.5+BIBN4096, $^{*}p$ =0.0203 <

0.05, pH 7.4 vs. LPS+R848, $^{*}p = 0.0112 < 0.05$, LPS+R848 vs. LPS+R848+BIBN4096, one-way ANOVA. n = 4 for *Asic3*$^{+/+}$ groups and n = 3 for *Asic3*$^{-/-}$ groups. Summary data are mean ± SEM.

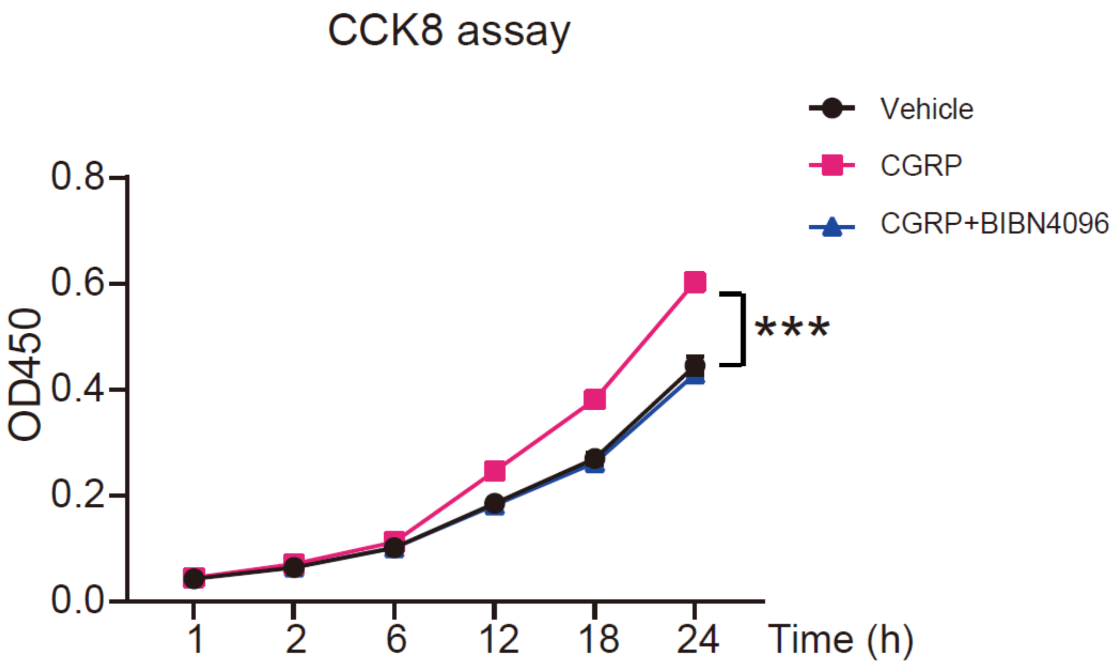

Supplementary Fig. 9 CGRP increases proliferation of HaCaT keratinocytes. Confluent and growth-arrested HaCaT keratinocytes were incubated with 100 nM CGRP or 100 nM CGRP plus 25 μM BIBN4096 and then subjected to CCK8 assay at time points indicated. $F_{(2,144)} = 163.0$, For 12 h, $^{***}p < 0.001$, vehicle vs. CGRP, $^{***}p < 0.001$, CGRP vs. CGRP+BIBN4096; For 18 h, $^{***}p < 0.001$, vehicle vs. CGRP, $^{***}p < 0.001$, CGRP vs. CGRP+BIBN4096; For 24 h, $^{***}p < 0.001$, vehicle vs. CGRP, $^{***}p < 0.001$, CGRP vs. CGRP+BIBN4096; two-way ANOVA. n = 9 for each group. Data are mean ± SEM and most errors are smaller than the symbols.

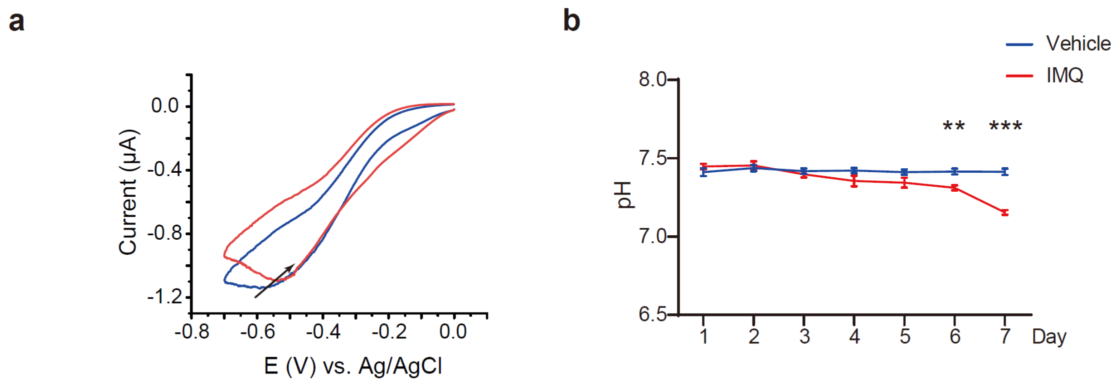

Supplementary Fig. 10 Psoriatic skin exhibits acidosis. a Representative cyclic voltammetry curves measured by Hemin-Fc/CNF microelectrode in the dorsal skins of vehicle (*blue*) and IMQ-treated (*red*) wild type mice, which demonstrated redox peaks at around –0.6 V and –0.5 V, respectively. The E (V) vs. Ag/AgCl value of $Fe^{2+/3+}$ in hemoglobin shifts during pH changes, as indicated by the black arrow. **b** pH values obtained in the vehicle or imiquimod-treated skin from *Asic3*$^{+/+}$ mice on consecutive days. $F_{(1,98)} = 31.40$, Day 6: $^{**}p = 0.0087 < 0.01$; Day 7: $^{***}p < 0.001$; vehicle vs. IMQ, two-way ANOVA. n = 8 for each group. Data are mean ± SEM.

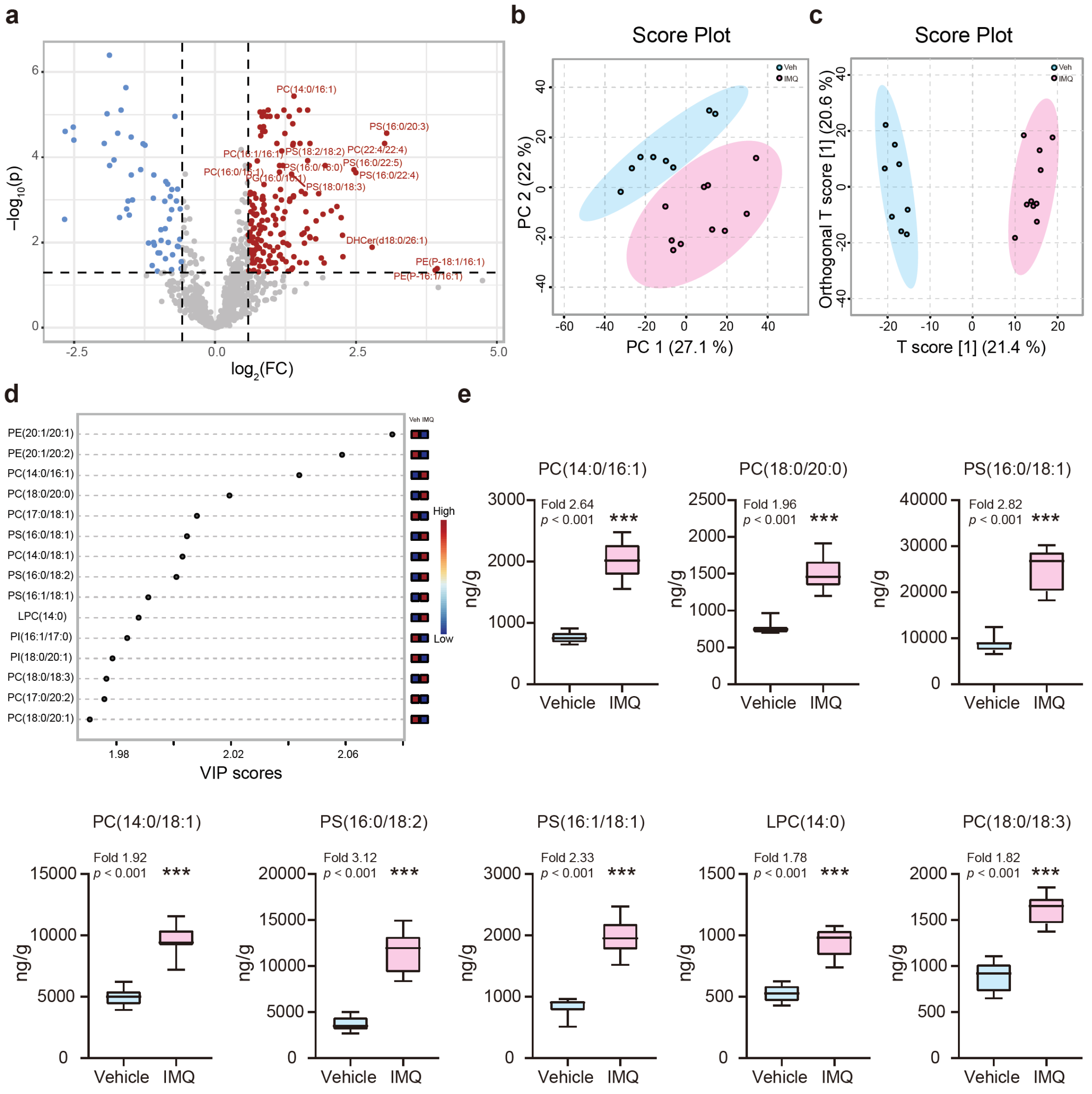

Supplementary Fig. 11 Psoriatic skin exhibits altered lipid profiles in lesional skin. **a** Log2-fold change in the normalized counts of various lipids in psoriatic skin vs. normal control (vehicle-treated). **b** Score plot of principal component analysis based lipidomic data of skins from psoriatic skin and normal control. **c** Score plot of orthogonal partial least squares discriminant analysis (OPLS-DA) distinguishing IMQ-treated and vehicle control based on tissue lipidomic profiling (R_2Y = 0.982, Q_2 = 0.914). **d** Variable importance in projection (VIP) scores for the top 15 lipids contributing to psoriatic inflammation. **e** Box-and-Whisker plots of altered lipids in IMQ-treated skin vs. vehicle control. Fold changes in peak intensity are indicated. $^{***}p$ < 0.001, two-tailed unpaired Student's *t* test. Whiskers represent minimum and maximum values.

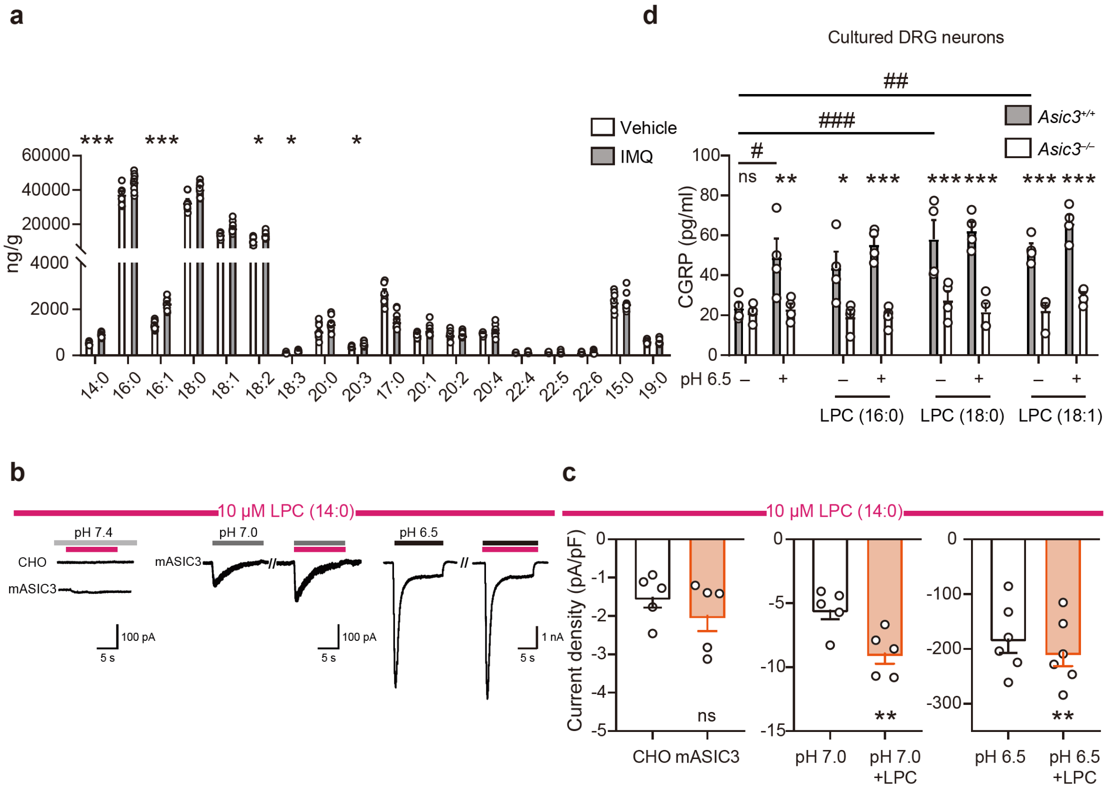

Supplementary Fig. 12 Variable involvement of different LPC molecular species in psoriatic inflammation. a LPC molecular species levels in skin samples of *Asic3*$^{+/+}$ mice treated with IMQ compared to vehicle control. The raw data were processed by probabilistic quotient normalization and autoscaling method and then analyzed by multiple comparisons with Benjamini-Hochberg adjustment. $F_{(1,304)}$ = 39.71, LPC (14:0): $^{***}p < 0.001$; LPC (16:0): $p = 0.9865$; LPC (16:1): $^{***}p < 0.001$; LPC (18:0): $p = 0.9176$; LPC (18:1): $p = 0.8310$; LPC (18:2): $^{*}p = 0.0277 < 0.05$; LPC (18:3): $^{*}p = 0.0368 < 0.05$; LPC (20:0): $p = 0.6176$; LPC (20:3): $^{*}p = 0.0285 < 0.05$); LPC (17:0): $p = 0.0629$; LPC (20:1): $p = 0.9820$; LPC (20:2): $p > 0.9999$; LPC (20:4): $p > 0.9999$; LPC (22:4): $p > 0.9999$; LPC (22:5): $p > 0.9999$; LPC (22:6): $p > 0.9999$; LPC (15:0): $p > 0.9999$; LPC (19:0): $p > 0.9999$; multiple comparisons with Benjamini-Hochberg adjustment. **b** Whole-cell currents recorded at –60 mV in CHO cells transfected with *mAsic3*. Bath solution was changed to pH 7.4, pH 7.0 and pH 6.5 containing LPC14:0 (10 µM) or not as indicated in the horizontal bars. **c** Peak current density without leak subtraction in response to exposure to bath solutions of different pH before and after applications of LPC14:0. pH 7.4: $p = 0.4608$, n = 5; pH 7.0: $^{**}p = 0.0072 < 0.01$, n = 5; pH 6.5: $^{**}p = 0.0081 < 0.01$, n = 6; paired t-tests. **d** CGRP levels in supernatant of DRG neurons from *Asic3*$^{+/+}$ and *Asic3*$^{-/-}$ mice exposed to pH 7.4 and pH 6.5 for 30 min without or with 30 µM LPC16:0, LPC18:0, or LPC18:1. n = 4 for each group. $F_{(1,48)}$ = 123.3, pH 6.5: $^{**}p = 0.0059 < 0.01$; *Asic3*$^{-/-}$ vs. *Asic3*$^{+/+}$; *Asic3*$^{+/+}$: $F_{(7,48)} = 4.616$, $p = 0.1985$, pH 7.4 vs. pH 7.4+LPC16:0; $^{***}p = 0.0005 < 0.001$, pH 7.4 vs. pH 7.4+LPC18:0; $^{**}p = 0.0053 < 0.01$, pH 7.4 vs. pH 7.4+LPC18:1; two-way ANOVA. Data are mean ± SEM.

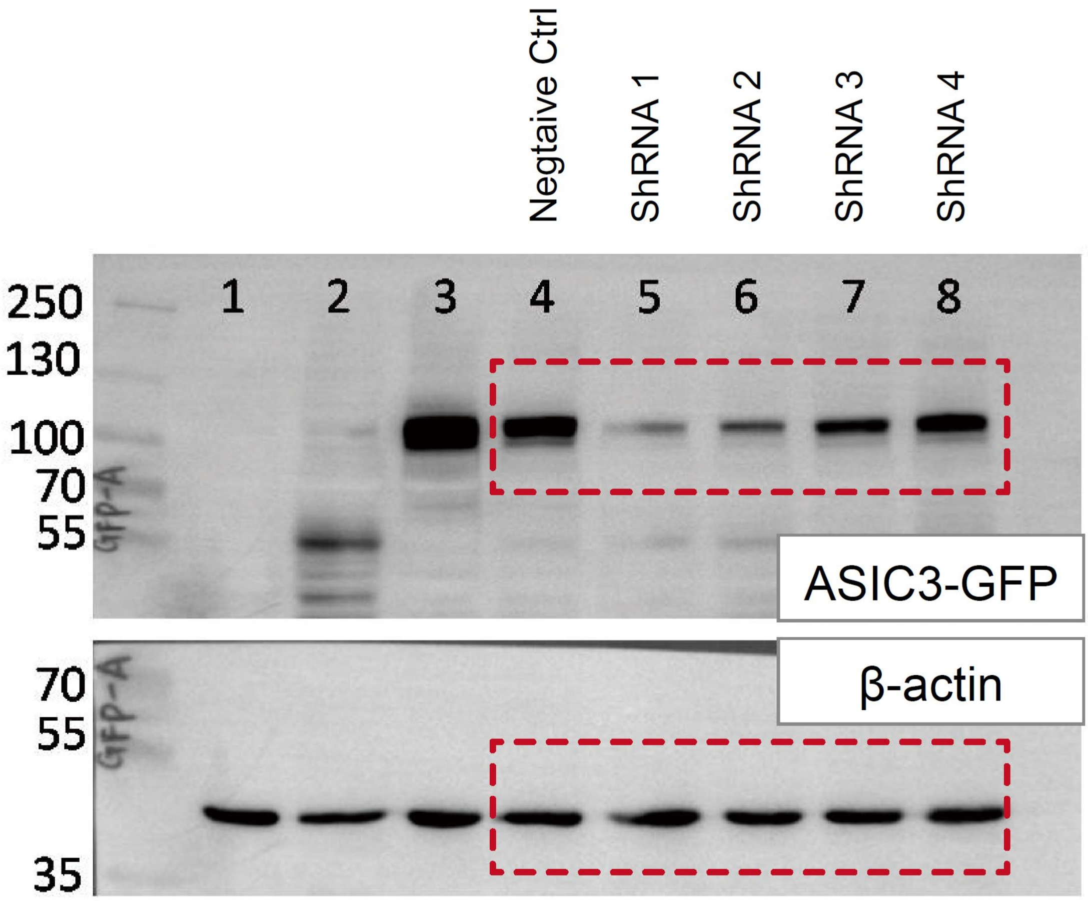

Supplementary Fig. 13 Uncropped images of Western blots shown in Supplementary Fig. 3c.
