# Supplementary material for: Sensory ASIC3 channel exacerbates psoriatic inflammation via a neurogenic pathway in female mice
Source: Nat Commun. 2024 Jun 20;15:5288. doi: 10.1038/s41467-024-49577-3 (PMC11190258; doi:10.1038/s41467-024-49577-3)
Supplement: Supplementary file 3 — Reporting Summary [file 41467_2024_49577_MOESM3_ESM.pdf]

nature portfolio

Corresponding author(s): Tian-Le Xu
Last updated by author(s): Apr 12, 2024

Reporting Summary

Nature Portfolio wishes to improve the reproducibility of the work that we publish. This form provides structure for consistency and transparency in reporting. For further information on Nature Portfolio policies, see our Editorial Policies and the Editorial Policy Checklist.

Statistics

For all statistical analyses, confirm that the following items are present in the figure legend, table legend, main text, or Methods section.

n/a	Confirmed	
☐	☒	The exact sample size (*n*) for each experimental group/condition, given as a discrete number and unit of measurement
☐	☒	A statement on whether measurements were taken from distinct samples or whether the same sample was measured repeatedly
☐	☒	The statistical test(s) used AND whether they are one- or two-sided<br>*Only common tests should be described solely by name; describe more complex techniques in the Methods section.*
☐	☒	A description of all covariates tested
☐	☒	A description of any assumptions or corrections, such as tests of normality and adjustment for multiple comparisons
☐	☒	A full description of the statistical parameters including central tendency (e.g. means) or other basic estimates (e.g. regression coefficient) AND variation (e.g. standard deviation) or associated estimates of uncertainty (e.g. confidence intervals)
☐	☒	For null hypothesis testing, the test statistic (e.g. *F*, *t*, *r*) with confidence intervals, effect sizes, degrees of freedom and *P* value noted<br>*Give P values as exact values whenever suitable.*
☒	☐	For Bayesian analysis, information on the choice of priors and Markov chain Monte Carlo settings
☒	☐	For hierarchical and complex designs, identification of the appropriate level for tests and full reporting of outcomes
☒	☐	Estimates of effect sizes (e.g. Cohen's *d*, Pearson's *r*), indicating how they were calculated

Our web collection on statistics for biologists contains articles on many of the points above.

Software and code

Policy information about availability of computer code

Data collection	pCLAMP 10.5 (Molecular Devices, USA)<br>Fluoview Confocal microscope FV3000 (Olympus, Japan)<br>BioTek Synergy HTX (Agilent, USA)<br>LSRFortessa (BD Biosciences, USA)<br>LightCycler 96 System (Roche, Switzerland)
Data analysis	FlowJo (version 10.10, BD Biosciences, USA)<br>GraphPad Prism (version 8.0.2, GraphPad Software, Inc., USA)<br>pCLAMP 10.5 (Molecular Devices, USA)<br>ImageJ (version 1.8.0, NIH, USA)<br>Office 2019 (Microsoft, USA)

For manuscripts utilizing custom algorithms or software that are central to the research but not yet described in published literature, software must be made available to editors and reviewers. We strongly encourage code deposition in a community repository (e.g. GitHub). See the Nature Portfolio guidelines for submitting code & software for further information.

Data

Policy information about availability of data

All manuscripts must include a data availability statement. This statement should provide the following information, where applicable:

- Accession codes, unique identifiers, or web links for publicly available datasets
- A description of any restrictions on data availability
- For clinical datasets or third party data, please ensure that the statement adheres to our policy

All data supporting the findings of this study are available within the paper and its Supplementary Information. Source data are provided with this paper.

Research involving human participants, their data, or biological material

Policy information about studies with human participants or human data. See also policy information about sex, gender (identity/presentation), and sexual orientation and race, ethnicity and racism.

Reporting on sex and gender	No human participants or human data.
Reporting on race, ethnicity, or other socially relevant groupings	No human participants or human data.
Population characteristics	No human participants or human data.
Recruitment	No human participants or human data.
Ethics oversight	No human participants or human data.

Note that full information on the approval of the study protocol must also be provided in the manuscript.

Field-specific reporting

Please select the one below that is the best fit for your research. If you are not sure, read the appropriate sections before making your selection.

☒ Life sciences ☐ Behavioural & social sciences ☐ Ecological, evolutionary & environmental sciences

For a reference copy of the document with all sections, see nature.com/documents/nr-reporting-summary-flat.pdf

Life sciences study design

All studies must disclose on these points even when the disclosure is negative.

Sample size	We sought analogous studies in existing literature and selected sample sizes employed in those investigations (e.g. Lou F, et al. Immunity. 2020) . Additionally, a pilot study was undertaken prior to formal experiments, revealing that the standard deviation in our designs closely aligned with the published data.
Data exclusions	No data were excluded from the analyses.
Replication	Yes; the experiments were replicated for 3 times independently and all attempts at replication were successful.
Randomization	Randomization was used to assign all experimental groups without bias based on any covariates.
Blinding	All experiments were conducted using a double-blind approach for data collection and statistical analysis.

Reporting for specific materials, systems and methods

We require information from authors about some types of materials, experimental systems and methods used in many studies. Here, indicate whether each material, system or method listed is relevant to your study. If you are not sure if a list item applies to your research, read the appropriate section before selecting a response.

Materials & experimental systems

n/a	Involved in the study	
	☒	Antibodies
	☒	Eukaryotic cell lines
☒		Palaeontology and archaeology
	☒	Animals and other organisms
☒		Clinical data
☒		Dual use research of concern
☒		Plants

Methods

n/a	Involved in the study	
☒		ChIP-seq
	☒	Flow cytometry
☒		MRI-based neuroimaging

Antibodies

Antibodies used	Primary antibodies:<br>Anti-Cytokeratin 5 antibody (Abcam, Cat#ab52635, dilution 1:500)<br>Anti -Ki67 Mouse mAb (Servicebio, Cat#GB121141-100, dilution 1:500)<br>Alexa Fluor 700 anti-mouse CD45 antibody (BioLegend, Cat#103128, dilution 1:200)<br>PE-Cyanine 7 conjugated CD11c antibody (Invitrogen, Cat#25-0114-82, dilution 1:200)<br>FITC conjugated MHC II antibody (Invitrogen, Cat#11-5321-85, dilution 1:200)<br>PerCP conjugated IL-23 antibody (Invitrogen, Cat#46-7023-82, dilution 1:100)<br>Recombinant Anti-MYC Tag Mouse mAb (Servicebio, Cat# GB15076-100, dilution 1:500)<br>CGRP (D5R8F) Rabbit mAb (Cell Signaling Technology, Cat#14959, dilution 1:200)<br>Anti PGP9.5 antibody (Abcam, Cat#ab108986, dilution 1:200)<br>Anti-TRPV1 (VR1) antibody (Alomone Labs, Cat #ACC-029, dilution 1:200)<br>Secondary antibodies:<br>Donkey anti-Rabbit IgG (H+L) Highly Cross-Adsorbed Secondary Antibody, Alexa Fluor™ 488 (Thermo Fisher Scientific, Cat#A21206)<br>Donkey anti-Mouse IgG (H+L) Highly Cross-Adsorbed Secondary Antibody, Alexa Fluor™ 568 (Thermo Fisher Scientific, Cat#A10037)
Validation	All the antibodies used in the study were commercially available and the more information is available in the manufacturer website. Commercial antibodies were validated by vendor according to industry standards (as inserted below).<br>Abcam: Knock-out (KO) validation is a robust technique used to confirm antibody specificity by testing the antibody of interest in a KO cell line or tissue in which the target gene has been edited so that the target protein is not expressed. A specific antibody will detect the specific target protein signal in the unedited, wild-type (WT) cell line and yield no signal when tested in a KO cell line. In this way, KO validation serves as a true negative control to confirm antibody specificity to the protein of interest. At Abcam, we use an extensive library of human KO cell lines to validate our antibodies. Our KO cell lines are generated via CRISPR-Cas9 and editing is verified by Sanger sequencing or NGS. We also employ proteomic validation, where possible.<br>Biolegend (human and mouse) Flow Cytometry Reagents:-Specificity testing of 1-3 target cell types with either single- or multi-color analysis (including positive and negative cell types). Once specificity is confirmed, each new lot must perform with similar intensity to the in-date reference lot. Brightness (MFI) is evaluated from both positive and negative populations. Each lot product is validated by QCtesting with a series of titration dilutions. https://www.biolegend.com/en-us/quality/quality-control<br>Invitrogen: All antibodies on the Thermo Fisher Scientific website that have undergone and passed the advanced verification testing are identified with an "Advanced Verification" badge. The badge can be found in the antibody search results and at the top of the product detail pages. Data supporting the antibody's advanced verification status can be found in the data galleries on the product pages as well.<br>Cell Signaling Technology: To ensure our antibodies will work in your experiment, we adhere to the Hallmarks of Antibody Validation, six complementary strategies that can be used to determine the functionality, specificity, and sensitivity of an antibody in any given assay. CST adapted the work by Uhlen, et. al., ("A Proposal for Validation of Antibodies." Nature Methods (2016)) to build the Hallmarks of Antibody Validation, based on our decades of experience as an antibody manufacturer and our dedication to reproducible science.

Eukaryotic cell lines

Policy information about cell lines and Sex and Gender in Research

Cell line source(s)	Human Keratinocytes Cells (HaCaT)<br>Chinese hamster ovary cell K1 (CHO-K1)<br>Primary cultured DRG neurons (from female mice)<br>Primary cultured BMDCs (from female mice)
Authentication	The cells lines were authenticated using western blot and Sanger sequencing.
Mycoplasma contamination	The cells tested negative for micoplasma and were tested every two months.
Commonly misidentified lines<br>(See ICLAC register)	No commonly misidentified cell lines were used in this study.

Animals and other research organisms

Policy information about studies involving animals; ARRIVE guidelines recommended for reporting animal research, and Sex and Gender in Research

Laboratory animals	All experiments were performed on C57BL/6 mice obtained from Shanghai Slac Laboratory Animal Company, China. To minimize experimental variability, age-matched littermate pairs resulting from heterozygous crossings were used for all experiments. Asic3–/– mice were prepared as previously described in Cell Reports 13:387-398, 2015. To achieve nociceptor-specific deletion of Asic3, NaV1.8-Cre mice were bred with Asic3flox/flox mice. The NaV1.8-Cre mice were generously provided by Professor Xu Zhang at Shanghai Advanced Research Institute, Chinese Academy of Sciences, Shanghai, China and the Asic3flox/flox mice were provided by Professor Chih-Cheng Chen at Institute of Biomedical Sciences, Academia Sinica, Taipei, Taiwan. We generated Asic3-myc mice by CRISPR/Cas-mediated genome engineering. The 3xMyc tag was inserted downstream of ATG start codon in exon 11, where the mouse Asic3 gene located. All experiments were performed using 6-week-old female mice. All mice were bred in specific pathogen-free laboratory animal facilities under standard conditions with temperatures of 21-23 C, 40-60% humidity and 12 h light/dark cycles, with rodent chow and water ad libitum. Animal care and experimental protocol were approved by the Animal Ethics Committee of Shanghai Jiao Tong University School of Medicine, Shanghai, China.
Wild animals	The study did not involve wild animals.
Reporting on sex	Given the focus of our study on psoriasis, and considering the known sex-specific differences in disease onset and severity, we have deliberately chosen to include only female mice. This decision is supported by previous studies in the field (eg. Nature Communications, 7:13466, 2016; Journal of Clinical Investigation, 128(6):2551-2568, 2018) and aims to reduce variability in our data, enhancing the study's ability to detect meaningful effects. While this approach allows us to address our primary research question effectively, we acknowledge that it may limit the generalizability of our findings to both sexes, and we provide this contextual information with transparency and scientific rigor.
Field-collected samples	The study did not involve samples collected from the field.
Ethics oversight	All of the animal procedures were approved by the Animal Ethics Committee of Shanghai Jiao Tong University School of Medicine by the Institutional Animal Care and Use Committee (Department of Laboratory Animal Science, Shanghai Jiao Tong University School pf Medicine; Policy Number DLAS-MP-ANIM. 01-05).

Note that full information on the approval of the study protocol must also be provided in the manuscript.

Plants

Seed stocks	*Report on the source of all seed stocks or other plant material used. If applicable, state the seed stock centre and catalogue number. If plant specimens were collected from the field, describe the collection location, date and sampling procedures.*
Novel plant genotypes	*Describe the methods by which all novel plant genotypes were produced. This includes those generated by transgenic approaches, gene editing, chemical/radiation-based mutagenesis and hybridization. For transgenic lines, describe the transformation method, the number of independent lines analyzed and the generation upon which experiments were performed. For gene-edited lines, describe the editor used, the endogenous sequence targeted for editing, the targeting guide RNA sequence (if applicable) and how the editor was applied.*
Authentication	*Describe any authentication procedures for each seed stock used or novel genotype generated. Describe any experiments used to assess the effect of a mutation and, where applicable, how potential secondary effects (e.g. second site T-DNA insertions, mosiacism, off-target gene editing) were examined.*

Flow Cytometry

Plots

Confirm that:

- [x] The axis labels state the marker and fluorochrome used (e.g. CD4-FITC).
- [x] The axis scales are clearly visible. Include numbers along axes only for bottom left plot of group (a 'group' is an analysis of identical markers).
- [x] All plots are contour plots with outliers or pseudocolor plots.
- [x] A numerical value for number of cells or percentage (with statistics) is provided.

Methodology

Sample preparation	Single-cell suspensions were pelleted and resuspended in PBS with 2% FBS containing fluorophore-conjugated antibodies. Cells were initially stained with antibodies targeting cell surface proteins for 30min on ice and washed with PBS containing 2% FBS. For intracellular target staining, cells were then fixed and permeabilized using a Cytofix/Cytoperm kit (BD Biosciences, cat: 554714).<br>The following antibodies were used for mouse flow cytometry:<br>Alexa Fluor 700 anti-mouse CD45 antibody (BioLegend, Cat#103128)<br>PE-Cyanine 7 conjugated CD11c antibody (Invitrogen, Cat#25-0114-82)

	FITC conjugated MHC II antibody (Invitrogen, Cat#11-5321-85)<br>PerCP conjugated IL-23 antibody (Invitrogen, Cat#46-7023-82)
Instrument	Cells were acquired on BD LSRFortessa. Raw data were analyzed with FlowJo (BD).
Software	Raw data were analyzed with FlowJo (version 10.10, BD Biosciences, USA)
Cell population abundance	A total of 10,000 positive cells were sorted per sample. The purity of the sorted population was evaluated post-sorting by re-analyzing, consistently demonstrating a purity level exceeding 99%.
Gating strategy	For the examination of BMDCs response during co-culture with DRG neurons, we employed surface markers CD45, CD11c, and MHC II to identify BMDCs (CD45+CD11c+MHC II+) isolated from the culture media.

[x] Tick this box to confirm that a figure exemplifying the gating strategy is provided in the Supplementary Information.
